# Multifocal Osteonecrosis Secondary to Chronic Alcohol Ingestion

**DOI:** 10.1155/2015/137273

**Published:** 2015-11-05

**Authors:** Kazu Matsumoto, Hiroyasu Ogawa, Haruhiko Akiyama

**Affiliations:** Department of Orthopaedic Surgery, Gifu University School of Medicine, 1-1 Yanagido, Gifu, Gifu 501-1194, Japan

## Abstract

Multifocal osteonecrosis is a relatively rare disorder with an estimated incidence of around 3% among patients diagnosed as having osteonecrosis. Multifocal osteonecrosis is caused by the several conditions including corticosteroid treatment, coagulation disorders, connective tissue disorders including systemic lupus erythematosus (SLE), inflammatory bowel disease, renal transplantation, and underlying malignancies. Alcohol abuse is one of the risk factors for osteonecrosis, and alcohol-induced osteonecrosis is 5% among all the osteonecrosis. Furthermore, the overall incidence of alcohol-induced multifocal osteonecrosis was approximately 6% among all the osteonecrosis induced by the alcohol. Therefore, here, we report an extremely rare case of alcohol-induced multifocal osteonecrosis involving three joints (two knees and one hip) and review the related literature.

## 1. Introduction

Osteonecrosis is believed to be the result of ischemia of the juxta-articular bone. Multifocal osteonecrosis is defined as disease affecting three or more joints, and its overall incidence is estimated to be around 3% among patients diagnosed as having osteonecrosis [1]. The risk factors associated with multifocal osteonecrosis are corticosteroid treatment [1], alcohol abuse [2, 3], coagulation disorders [4], connective tissue disorders including systemic lupus erythematosus (SLE) [5], inflammatory bowel disease [6], renal transplantation [1], underlying malignancies [7], HIV infection [8], sickle cell disease [9], and trauma [10]. Although chronic alcohol intake is a common cause of osteonecrosis, multifocal osteonecrosis is relatively rare in this context. Orlić et al. [11] reported that the overall incidence of alcohol-induced multifocal osteonecrosis was extremely rare among all the osteonecrosis induced by the alcohol. Here we report a rare case of alcohol-induced multifocal osteonecrosis involving three joints (two knees and one hip) and review the related literature.

## 2. Case Report

**Figure 1:**
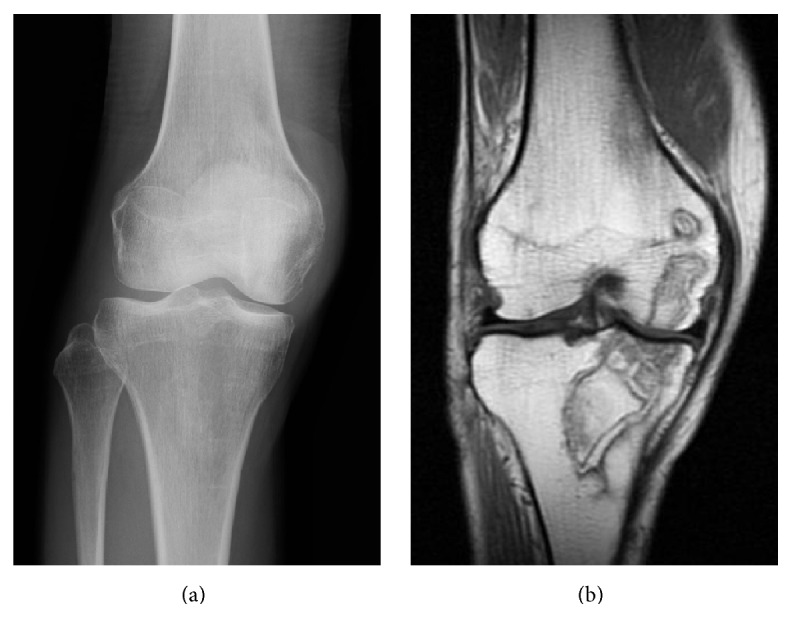
(a) Anteroposterior radiograph of the right knee showing increased radiodensity of the proximal half of the tibia. (b) T1W coronal section MRI showing large demarcated regions of abnormal signal intensity in the proximal half of the tibia and medial femoral condyle, with features suggestive of osteonecrosis.

**Figure 2:**
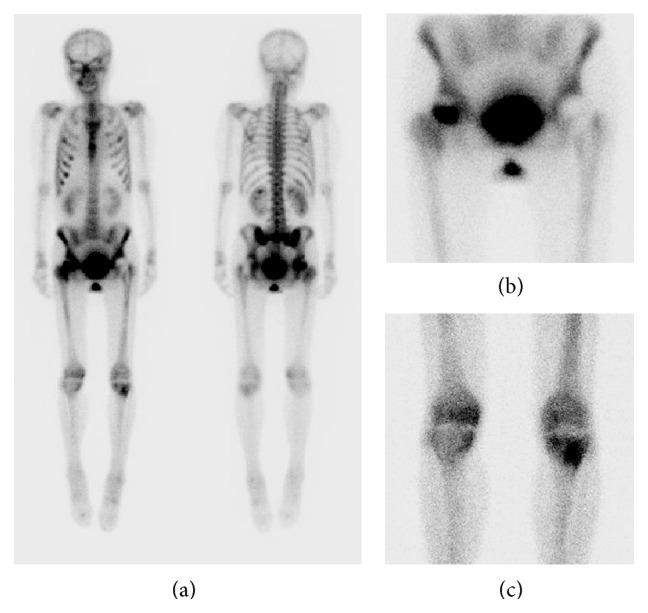
Bone scintigram showed the abnormal uptake in the right hip (a, b) and the right and the left knee joints (a, c).

**Figure 3:**
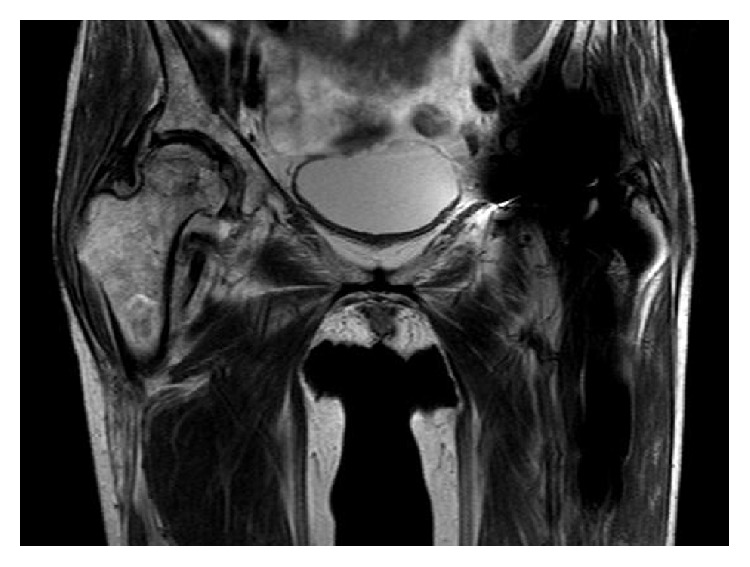
T1W mid-coronal section MRI showing osteonecrosis of the right femoral head.

**Figure 4:**
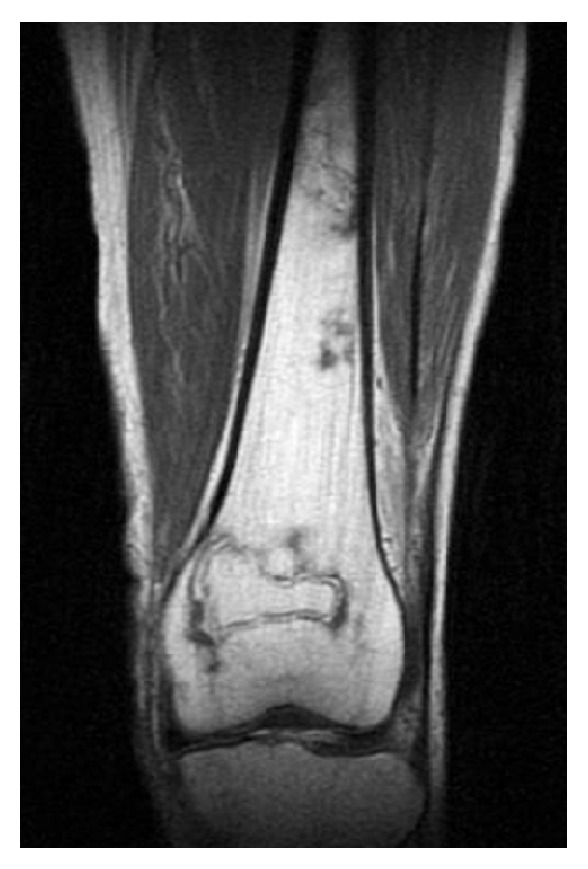
T1W coronal section MRI showing multiple demarcated bone marrow abnormalities with double-line sign, compatible with osteonecrosis.

A 47-year-old woman presented with a complaint of generalized right knee pain. Physical examination of the right knee joint revealed nothing remarkable. The patient had a history of chronic alcohol consumption exceeding 400 mL of ethanol per week for the past 20 years. There was no history of steroid intake or trauma. One year before presentation, she had undergone a bipolar hip replacement for avascular necrosis of the left hip. She referred to our hospital due to the right knee pain. Radiographs revealed no destruction of the articular surfaces of the right knee, but increased radiodensity was evident in the proximal half of the tibia extending from the joint line (Figure 1(a)). MRI demonstrated large demarcated regions of abnormal signal intensity in the proximal half of the tibia and medial femoral condyle, with features of osteonecrosis (Figure 1(b)). A bone scintigram showed the abnormal uptake in the right hip, and the right and the left knee joints (Figures 2(a), 2(b), and 2(c)). A full blood screen including autoantibodies, a clotting profile, ALP, and AMY gave normal results (Table 1). Human immunodeficiency virus (HIV) and hepatitis B and C viruses and antibodies were negative. The patient complained that she had often felt mild pain in her right hip and left knee joint after a short walk. Physical examination of the right hip and left knee revealed no abnormality. However, radiographs demonstrated increased radiolucency in both, and MRI revealed demarcated regions of abnormal signal intensity in the central portion of the right femoral head with typical features of osteonecrosis (Figure 3). Left knee MRI showed multiple demarcated bone marrow abnormalities with double-line sign in the distal shaft of the left femur, compatible with bone necrosis (Figure 4).

The patient was instructed to maintain only partial weight-bearing with wheelchair mobilization for eight weeks and then to return gradually to full weight-bearing over the next two months. Eleven months after the initial presentation, she complained of mild pain in her right hip. Plain radiograph revealed the collapse of her right femoral head. A bipolar hip arthroplasty was performed on the right side. A biopsy of the femoral head confirmed the diagnosis of osteonecrosis of the femoral head. Thereafter, no symptoms were evident in both knee joints. Abstinence from alcohol was also achieved successfully.

## 3. Discussion

Multifocal osteonecrosis is a relatively rare disorder with an estimated incidence of around 3% among patients diagnosed as having osteonecrosis [1]. The Collaborative Osteonecrosis Group Study has confirmed that the primary cause of multifocal osteonecrosis is a history of corticosteroid therapy [1]. The most common conditions that require this form of treatment are systemic lupus erythematosus (SLE), renal transplantation, inflammatory bowel disease, and coagulation disorder [1, 4–6]. Our patient showed no abnormalities in blood examinations including autoantibodies and clotting profile. Excess alcohol consumption was the only problem.

The hip joint is the site most commonly affected by multifocal osteonecrosis, followed by the knee, shoulder, ankle, elbow, and wrist [1]. Bilaterality is common, including 98% of affected hips, 87% of affected knees, and 83% of affected shoulders [1]. Our patient had definite osteonecrosis affecting three anatomical sites, the bilateral knees and right hip, and a bone scan of the humeral heads was negative (data not shown). Thus, the features were typical of multifocal osteonecrosis.

The overall incidence of multifocal osteonecrosis induced by alcohol ingestion is approximately 6% [11]. There have been only two detailed reports of alcohol-induced multifocal osteonecrosis. Moon et al. [2] reported a case involving both hips and both knees with a 14-year follow-up, and Roach et al. [3] reported a case affecting the hip and both knees with intramedullary bone infarction in both the distal femur and proximal tibia. The exact levels of alcohol intake were not defined. The Japanese Orthopaedic Association threshold for osteonecrosis is 400 mL of ethanol per week [12]. These previous cases and the present one satisfied this condition. Although, overall, alcohol-induced multifocal osteonecrosis is rare, attention should be paid to the possibility of osteonecrosis at multiple anatomic sites.

Fortunately, our patient continues to be asymptomatic with conservative treatment. However, the other two reported cases of alcohol-induced multifocal osteonecrosis were treated by total joint arthroplasty [2, 3]. The Collaborative Osteonecrosis Group Study confirmed that the most common treatment was core decompression in 46% of affected hips and 47% of affected knee joints [1]. Total joint arthroplasty was performed in 43% of affected hips, and 21% of affected knees. Because of the high rate of surgical treatment, multifocal osteonecrosis needs to be managed carefully, including monitoring of abstinence from alcohol.

Although multifocal osteonecrosis secondary to chronic alcohol ingestion is rare, if the condition is suspected, then proper imaging including MRI is necessary for clarifying the possible involvement of commonly affected anatomical sites, owing to the subclinical nature of its pathology.

## Figures and Tables

**Table 1:** Results of the blood examination.

Complete blood count		
WBC	4080/*μ*L	(3300–7900)
RBC	370 × 10^4^/*μ*L	(369–507)
Hb	12.7 g/dL	(11.3–15.4)
Ht	38.7%	(34.0–46.3)
Plt	26 × 10^4^/*μ*L	(15.5–35.0)

C-reactive protein	<0.07 mg/dL	<0.20
ESR	8 mm/h	

Immunological test		
Anti-DNA	Negative	
Antiphospholipid Ab	Negative	

Thrombophilia screen		
Antithrombin III activity	95%	(80–130)
Protein C activity	132%	(64–146)
PT	12.6 s	(9.5–13.5)
APTT	28.6 s	(25.0–43.0)
Fibrinogen	211 mg/dL	(150–350)
D-dimer	<0.7 *μ*g/mL	(<1.0)
